# Chronic kidney disease in cancer patients, the analysis of a large oncology database from Eastern Europe

**DOI:** 10.1371/journal.pone.0265930

**Published:** 2022-06-09

**Authors:** Mircea Ciorcan, Lazar Chisavu, Adelina Mihaescu, Florica Gadalean, Flaviu Raul Bob, Serban Negru, Oana Marina Schiller, Iulia Dana Grosu, Luciana Marc, Flavia Chisavu, Razvan Dragota Pascota, Adrian Apostol, Viviana Ivan, Adalbert Schiller

**Affiliations:** 1 Department of Clinical Practical Skills, Victor Babes University of Medicine and Pharmacy, Timisoara, Romania; 2 Center of Advanced Research in Cardiovascular Pathology and Hemostaseology, “Victor Babes” University of Medicine and Pharmacy, Timisoara, Romania; 3 Division of Nephrology, Dept. of Internal Medicine II, “Victor Babeș” University of Medicine and Pharmacy, Timisoara, Romania; 4 Centre for Molecular Research in Nephrology and Vascular Disease, Faculty of Medicine, “Victor Babeș” University of Medicine and Pharmacy, Timișoara, Romania; 5 County Emergency Hospital Timisoara, Timisoara, Romania; 6 Oncohelp Medical Center Timisoara, Oncology, Timisoara, Romania, “Victor Babeș” University of Medicine and Pharmacy, Timișoara, Romania; 7 Avitum BBraun Romania Timisoara, Nephrology, Timisoara, Romania; 8 Emergency Hospital for Children Louis Turcanu Timisoara, Pediatric Nephrology, “Victor Babeș” University of Medicine and Pharmacy, Timișoara, Romania; 9 County Emergency Clinical Hospital, Nephrology, Timisoara, Romania; 10 County Emergency Clinical Hospital, Cardiology, Timisoara, Romania; 11 Division of Cardiology, Dept. of Internal Medicine II, "Victor Babeș" University of Medicine and Pharmacy, Timisoara, Romania; University of Glasgow, UNITED KINGDOM

## Abstract

**Introduction:**

Kidney dysfunction is prevalent in oncology patients and has an impact on their treatment and quality of life. The aim of our study was to analyze the prevalence of CKD in a large cohort of several types of cancer patients in an East European Region.

**Material and methods:**

We conducted an observational retrospective cohort study on 5831 consecutive, biopsy-diagnosed cancer patients between January 2019 –December 2020 in the largest oncology hospital and outpatient clinic in Western Romania. 4342 subjects were included in the statistical analysis.

**Results and discussion:**

From the 24 cancer types, the most prevalent cancers were represented by: breast (22.02%), lung (10.18%) and colonic cancer (9.51%). The prevalence of CKD (G3 –G5) was 12.27% after the first year of follow-up and 13.42 after the second year. The prevalence of CKD was higher in patients with renal (50%), urinary tract (33.6%) and pancreatic cancers (19.6%) and lower in patients with colonic cancers (5.3%) and brain tumors (2.5%). At the end of our 2-year survey period, 0,7% of the CKD cases had an eGFR around 6 ml/min/1.73m^2^ –an indication for renal replacement therapy.

**Conclusion:**

Oncology patients have a significantly higher prevalence of CKD compared to the general population, dependent of the age of the patients and the type of cancer. The prevalence of advanced CKD was surprisingly high (stages G4-G5 Pre-Dialysis 22.15%) one third of the CKD- G5 patients having indication for initiation of renal replacement therapy. An onco- nephrology team should be needed for the best medical care of these patients.

## Introduction

Chronic kidney disease and cancers have complex and intricate relation. Frequently the age of patients at the cancers onset is more advanced, when kidney function may already be decreased due to hypertension, diabetes mellitus or a normal ageing process. Under these conditions, chronic kidney disease (CKD) may precede the onset of cancer and may act as risk factor for its development.

At the same time, neoplasms represents a risk factor for CKD due to the repeated aggressive and potential nephrotoxic therapeutic interventions, malnutrition, hyperuricemia, paraneoplastic syndrome involving the kidney and the cancer per se. In different countries, in cohorts of patients with solid tumors the prevalence of CKD stage G3 and up varies between 12% (France) and 25% (Japan) [1].

The various nephrotoxic effects of drugs and cancer related comorbid condition might induce acute and/or chronic kidney damage i.e. acute kidney injury/disease (AKI/AKD) and/or CKD. Moreover, every episode of AKI/AKD is an important risk factor for CKD development [2, 3].

The novel therapies improve cancer patient’s survival and increase their possibility to reach advanced CKD stages or renal replacement therapy.

The emerging onco-nephrology sub-specialty tends to have a more holistic approach to these sophisticated and complicated issues [1].

The aim of our study was to assess the prevalence of CKD in a cohort of cancer patients. For this aim we analyzed the largest and most representative cancer database from Western Romania.

## Material and methods

A large oncology data base from Western Romania was analyzed in this study. A total number of consecutive 5831 biopsy-diagnosed cancer patients between January 2019 –December 2020 in the largest oncology hospital and outpatients clinic in Western Romania, were included. The study design was observational retrospective cohort study.

All patients included in this study underwent some form of cancer treatment (chemotherapy, radiotherapy and/or surgery) prior or after inclusion in the database. At the inclusion all patients signed an informed consent for the use of the registered medical and personal data for scientific purposes under the condition of strict anonymity. The identity of the patients was blinded for the investigators by a computer assigned code. The study was approved by the Ethic Comity for Research of the authors university.

Serum creatinine levels were measured via in venous blood samples at inclusion in order to estimate eGFR by the CKD-Epi formula [4]. During the study period 2-year, serum creatinine was repeatedly determined in order to estimate GFR (at least 2 determinations). The patients without at least 2 serum creatinine measurements in the 2 years period, have been excluded from the study. Missing data, uncertain cancer diagnosis and rare/not common types of cancer for the study population have also been excluded (1489 patients). The remaining 4342 patients were included in our evaluation (average age 62.5 +/- 10.3 years, 56.3% female). Acute kidney injury and Acute kidney disease (i.e. increased serum creatinine/decreased eGFR normalized before 90 days) have not been included in the CKD statistics. We could not assess the effect of cancer therapy on kidney function since some of the interventions (chemo/radio therapy, surgery) have been performed in other medical units from neighboring counties and data were missing from the analyzed data base. CKD was defined as eGFR< 60ml/min/1.73m^2^, persistent for more than 90 days, according to 2012 KDIGO Guidelines [5]. Actually, the first registered serum creatinine determination was considered baseline creatinine. If eGFR was lower than 60ml/min/1.73m^2^ in this baseline GFR estimation and after 90 days or more (median 229 days– 7.6 months) eGFR was still below 60ml/min/1.73m^2^ the patient was considered having CKD and the value of the last GFR estimation was used for staging CKD. If the second eGFR was higher than 60ml/min/1.73m^2^ the patient was not considered to have CKD. We did not use albuminuria, proteinuria, and urine sediment as markers of chronic kidney damage since results may be biased by the possible toxic renal effects of the oncology treatment. For comparison, the 2008 CKD epidemiology data in the general population of Romania was used (same diagnosis criteria for CKD eGFR <60 ml/min/1.73 m^2^ for more than 3 months) [6].

### Statistical analysis

Continuous variables have been expressed as mean ± standard deviation (SD) or medians (interquartile ranges), while categorical variables have been expressed as percentages. Shapiro-Wilk tests of normality were applied which indicated the data were normally distributed. Univariate comparisons of baseline characteristics, using Student’s t-test or Chi-square test as appropriate, and binomial logistic regression analyses were performed for CKD prevalence in each group, Groups with a statistically significant correlation to CKD were analyzed by multivariate binomial logistic regression analysis and the model fit was assessed using the likelihood ratio test. All statistical tests have been 2-tailed and a p-value <0.05 was considered statistically significant. Statistical analyses were performed using the SPSS Version 26 (Chicago, IL, USA) software.

## Results

The 4342 patients with neoplasia have been grouped into 24 cancer types (groups) for a more convenient data assessment. In our data base the most prevalent cancers were represented by: breast cancer (22.02%), lung cancer (10.18%) and Ccolonic cancer (9.51%). “Table 1”.

**Table 1:** Prevalence of cancer types/groups in the assessed data base.

DIAGNOSIS	*N*	(%)	Average age (years)	Female gender (%)
	(4342)			
			Mean (SD)	
Breast cancer	956	22.02%	62.46(10.67)	100
Lung cancer	442	10.18%	62.81 (10.09)	31.20
Digestive tract cancers				
• Esophageal cancers	37	0.85%	63.70 (11.91)	29.70
• Gastric cancer	182	4.19%	63.52 (9.1)	37.36
• Colonic cancer	413	9.51%	62.27 (10.13)	50.60
• Rectal cancer	346	7.97%	61.85 (10.83)	40.10
• Pancreatic cancers	92	2.12%	63.55 (9.58)	44.50
• Liver and gall bladder cancers	60	1.38%	63.33 (10.54)	43.30
Renal and Urinary tract cancers				
• Renal cancers	80	1.84%	63.74 (10.85)	40.00
• Urinary tract	122	2.81%	63.34 (9.7)	35.20
• Prostate cancer	374	8.61%	63.2(9.7)	0.00
Genital cancers				
• Uterus cancer	352	8.11%	62.6 (9.2)	100.00
• Ovary cancers	100	2.30%	61.98 (10.68)	100.00
• Testicular cancers	27	0.62%	62.21 (11.71)	0
Malignant hematologic disorders				
• Lymphoproliferative diseases	108	2.49%	61.07 (12,89)	50.90
• Myelomas	25	0.58%	63.32 (7.7)	68.00
• Other Hematological cancers	163	3.75%	62.10 (10.55)	67.4
ENT (ear nose throat)	129	2.97%	69.64 (11,5)	23.50
Pharyngeal cancers	54	1.24%	62.79 (10.12)	27.70
Thyroid cancers	20	0.46%	60.10(9.62)	60.00
Brain tumors	80	1.84%	62.05 (10.38)	37.50
Skin cancers	71	1.64%	62.77 (9.34)	45.00
Sarcomas	36	0.83%	61.33 (12.73)	47.22
Other cancers	73	1.68%	63.21 (11.46)	33.3
TOTAL	4342			

The prevalence of CKD (G3 –G5) at the end of the study was 13.42% (582 patients) significantly higher if compared to the general population (8.8%) [6] (p<0.0001).

Patients with CKD were significantly older (66.06 +/-9.50 vs. 62 +/-10.35 years; (p <0.0001) and the prevalence of CKD was directly correlated with the age of the patients. The female gender was dominant but did not differ significantly between the groups (CKD, non-CKD 58.5% vs 55.9%—Chi square p = 0.226). The average eGFR in the CKD group was 42.39+/-14.32 ml/min/1.73 m^2^ (vs 91.43 +/-16.52 in the non-CKD patients). After the 2-year follow-up of the data, the patients with CKD have been classified into CKD categories according to eGFR. “Table 2.”

**Table 2:** CKD patients classified according to eGFR [5].

CKD categories	N	Prevalence
G3a	315	54.1%
G3b	138	23.7%
G4	99	17%
G5 (pre dialysis)	30	5.15%

**Fig 1:**
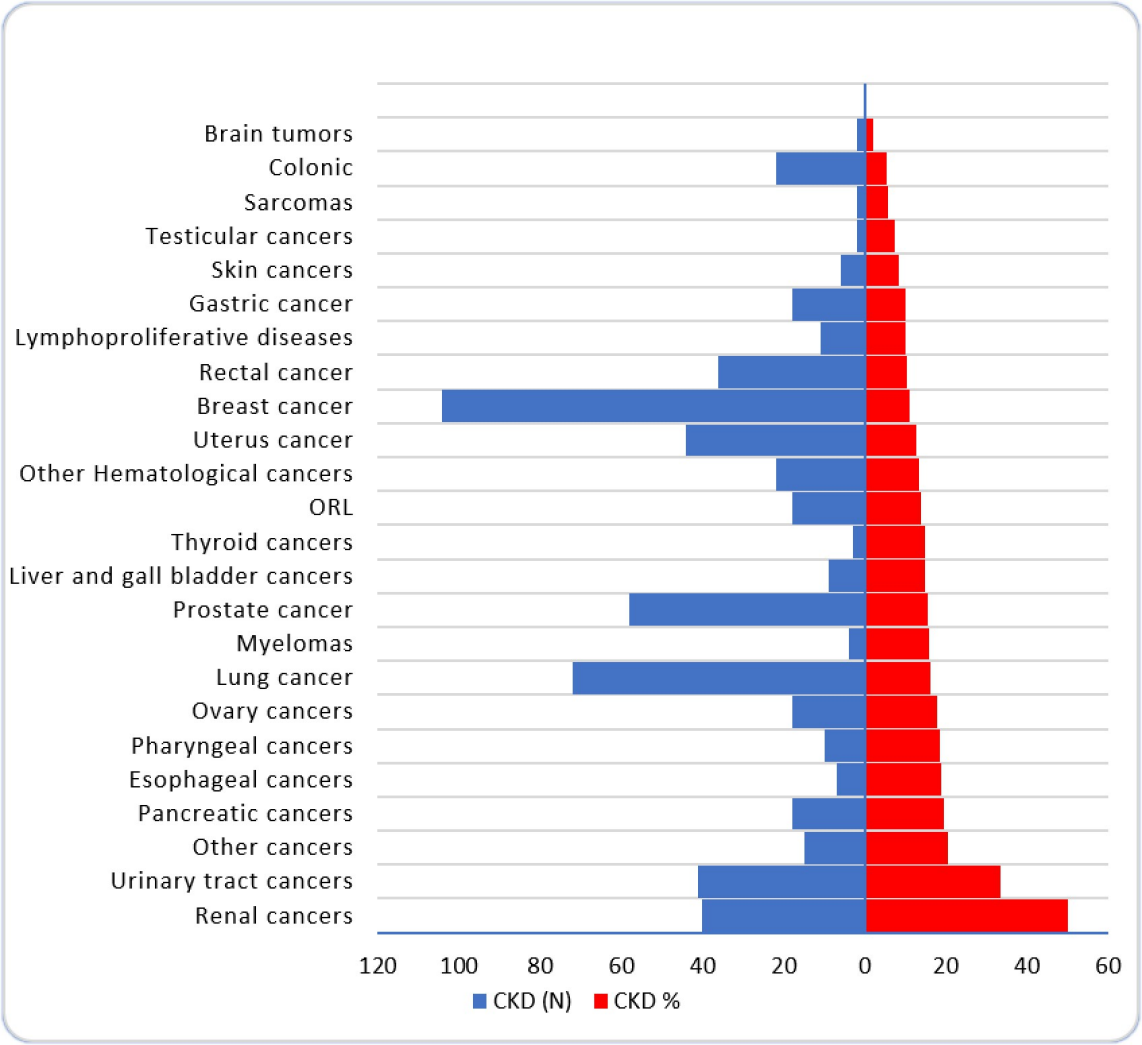
CKD prevalence and absolute number of CKD patients in different cancer types.

The prevalence of CKD was dependent of the type of cancer, being higher in patients with renal cancer (50%), urinary tract cancers (33.6%) and pancreatic cancers (19.6%). The prevalence of CKD was lower in patients with colonic cancers (5.3%) and brain tumors (2.5%). “Table 3” and “Fig 1”.

**Table 3:** Prevalence of CKD in different cancer types.

Cancer patients	N	CKD (N)	CKD %	CKD prevalence in cancer types compared to the cohort average,(p)
Renal cancers	80	40	50	< 0.0001***
Urinary tract cancers	122	41	33.6	< 0.0001***
Other cancers	73	15	20.5	0.059
Pancreatic cancer	92	18	19.5	0.067
Esophageal cancer	37	7	18.9	0.208
Pharyngeal cancer	54	10	18.5	0.233
Ovarian cancer	100	18	18	0.143
Lung cancer	442	72	16.2	0.059
Myelomas	25	4	16	0.658
Prostate cancer	374	58	15.5	0.170
Liver and gall bladder cancers	60	9	15	0.647
Thyroid cancer	20	3	15	0.790
ENT cancer	129	18	14	0.739
COHORT CKD PREV,			13.42	
Other Hematological cancers	163	22	13.4	0.881
Uterus cancer	352	44	12.5	0.788
Breast cancer	956	104	10.87	0.063
Rectal cancer	346	36	10.4	0.163
Lymphoproliferative diseases	108	11	10.1	0.375
Gastric cancer	182	18	9.8	0.206
Skin cancers	71	6	8.4	0.251
Testicular cancer	27	2	7.4	0.387
Sarcomas	36	2	5.7	0.187
Colonic cancer	413	22	5.3	< 0.0001***
Brain tumors	80	2	2.5	0.005***

Chi-square tests were made between every cancer type and the average prevalence of CKD of the studied cohort

*; **; *** Statistically significant differences (p<0,05; p<0,01 p<0,001)

In the more advanced CKD stages (G4-G5) kidney and urinary tract cancers are more frequent (23.2%), followed by digestive tract cancers (21.7%), breast cancers (15.5%) and lung cancers (10%). There were no significant differences concerning gender and age between the less severe CKD groups (G3a-G3b) and the more severe ones (G4-G5). In the G5 CKD group (30 cases), at the end of our 2-year study period, 33.3% of the cases had indication for initiation of renal replacement therapy (eGFR around 6 ml/min/1.73 m^2^). (For cutoff levels we used eGFR < 7ml/min/1.73m^2^, the upper value of late start hemodialysis patients used by the authors of The IDEAL study) [7].

In order to evaluate the risk of CKD in different types of cancer we used a multivariate logistic regression. All 24 types of cancer were included in the regression and Akaike information criteria (AIC) was used in order to determine the best model. In “Table 4” we present only the types of cancers that proved to be independent risk factor for CKD. In order to minimize bias, we adjusted our model for confounding factors (age, gender).

**Table 4:** Multiple logistic regression of cancer types for CKD.

Variable	*p*	Crude OR + 95% CI	*p*	Adjusted* OR + 95% CI
Pancreatic cancer	0.017	4.000(1.278–12.518)	0.024	3.766(1.194–11.880)
Other cancer	0.046	3.371(1.023–11.109)	0,058	3.202(0.961–10.665)
Pharyngeal cancer	0.003	6.024(1.845–19.670)	0,002	6.390(1.936–21.084)
Prostatic cancer	0.021	3.416(1.202–9.707)	0.010	4.019(1.399–11.546)
Renal cancer	<0.001	17.190(5.739–51.490)	<0.001	17.103(5.656–51.721)
Urinary tract cancer	<0.001	9.617(3.288–28.131)	<0.001	9.660(3.278–28.467)
Uterine cancers	0.015	3.671(1.292–10.435)	0.044	2.953(1.028–8.487)
Lung cancer	0.021	3.395(1.202–9.590)	0.020	3.471(1.222–9.864)

*adjusted for age and gender

The risk to develop CKD increases with the presence of cancer types from “Table 4”. Out of those types, renal, urinary tract and pharyngeal cancer present the highest risk to develop CKD. Renal cancer increases the risk by 17.19-fold, urinary tract cancers by 9.61-fold, pharyngeal cancer by 6.02-fold. After adjustment for age and gender the risk to develop CKD still remained high. (“Table 4”).

The influence of CKD on the survival of cancer patients could not be estimated due to the short follow-up time.

## Discussion

The complex bilateral relation between failing kidney function (acute or chronic) and cancers is more and more documented hence the need for a more complex approach. The onco-nephrology, by complex and intricate care and therapy, tend to more accurately fulfill the needs of patients with cancer and failing kidney function.

The prevalence of CKD in cancer patients was reported increased in several cross-section studies. In France, 2007, in a data base of 4684 cancer patients, CKD with GFR < 60ml/min/1.73m^2^ (MDRD formula) was identified in 12.02% of the patients with solid tumors (Stage 3 CKD was evidenced in 11.1% of the cases and stage 4 in 0.92%) [8]. In Austria 2014, the prevalence of CKD in a cohort of 1100 patients was 16.1% [9].

In our data base the prevalence of CKD was significantly higher as compared to the general population from Romania (13.4% vs.8.8% p<0.0001).

CKD patients were significantly older (66.06 +/-9.50 vs 62 +/-10,35 years; p <0.0001) and the prevalence of CKD was directly correlated with the age of the patients. Results were similar with the data reported by others [8]. Cancers and CKD occur with a higher rate in older ages. For example, in our data base (4342 patients) the mean age was 64 years, 33.9% of the patients were under 60 years of age and 66.1% were older than 60. Under 60 years of age the prevalence of CKD was 7.9% and over 60 the prevalence was more than 2-fold higher (16.2%). In many cases the kidney function is reduced prior to the cancer onset. The normal ageing of the kidney and the ageing related decrease of GFR is documented. In Caucasians over 60 years normal eGFR is around 75 ml/min/1.73m^2^ in men and 68 ml/min/1.73m^2^ in women and in people of Japanese ethnicity is 73 ml/min/1.73m^2^ [10, 11]. Preexisting hypertension and type 2 DM may further decrease kidney function prior to cancer onset. So older persons, if develop cancer, may already present a reduced renal functional reserve and that may influence treatment and care. Reduced renal reserves are risk factors for CKD and AKI also [12].

On the other hand, CKD may represent a risk factor for the development of cancers but the existing data are controversial. In moderate CKD (eGFR < 55 ml/min/1.73m^2^) the risk of cancer was considered to increase by 40% in men (2009) [13]. A 2016 meta-analysis however, did not confirm these findings. Still, the increased risk in end stage renal disease (ESRD) and in transplant patients was documented [14]. In 2018 the increased risk of colorectal cancers was evidenced in both pre dialysis CKD and in organ transplant recipient patients [15]. Increased risk of nonmelanoma skin cancers in Stage 5 pre dialysis patients [16], urinary tract cancers and hematologic malignancies, in all CKD patients, have been signaled also [17]. It is not very clear how advanced CKD increases the risk of cancer but uremic toxins, increased prevalence of common risk factors (for cancer and CKD), cytotoxic and immunologic agents used for the treatment of glomerular and tubulointerstitial diseases have been postulated to contribute. The chronic treatment with different immunomodulators used in transplants to improve transplanted organ survival may also increase the risk of cancer [13, 17, 18].

The risk of CKD in cancer patients may be related to the type of cancers and the organ involved as well as the specific cancer therapies indicated in the guidelines and the repeated treatment sessions [1]. One should remember also that most of the cancer patients (having an age around 60 years) present comorbid conditions and complications and receive potential nephrotoxic therapies. The risk factors for drug induced nephrotoxicity are multiple and are frequently associated in the same cancer patient (advanced age, hypovolemia, CKD, nephrotoxic drug associations, hypoalbuminemia, cardiovascular disease, genetic polymorphisms, diabetes, obesity, high dose hypotensive agents). The most frequent classical nephrotoxic inductors of AKI/AKD and CKD are represented by nonsteroidal anti-inflammatory drugs (NSAIDs), antibiotics/ antivirals (Vancomycin, Aminoglycosides, Polymyxins, Amphotericin B, Tenofovir), Cisplatin, Methotrexate, Calcineurin inhibitors, and so on [19]. It is interesting to mention the fact that even in critically ill patients (on high risk for nephrotoxic damage), in critical care units (medical staff with high awareness of the renal damage risk) 22.52% of the drugs prescribed were potentially nephrotoxic [20]. Classical and novel anti-cancer immunotherapies (Interferon alpha, Interleukin-2, Immune checkpoint inhibitors, the emerging CAR-T cell therapy) have potential nephrotoxic effects also due to their secondary immune effects [21]. The cancer patients in our data base received specific treatment according to international guidelines. However, the data base did not record the number of treatment sessions for relapses or the number of AKI episodes, therefore, we didn’t assess the drug related CKD epidemiology

In our data base the prevalence of cancer types is dominated by: breast, lung, colonic and prostate cancer (see also “Table 1”). The prevalence/incidence of cancer types may differ in local data bases as compared to general statistics [14]. The prevalence of CKD differed related to cancer types. If patient specific and treatment specific risk/initiating factors for CKD have been mentioned earlier, one should not forget the cancer specific risk factors which could influence the incidence/prevalence of CKD. Paraneoplastic syndromes involving glomerular, tubulointerstitial and vascular compartments of the kidney, fluid-electrolyte disorders, tumor infiltration, urinary obstruction may represent important initiating factors for CKD development [1]. In our data base the prevalence of CKD was higher as compared to the average prevalence at 2 years (13.42%) in 13 of the cancer groups but the differences were reaching statistical significance only in renal cancers (50%, p<0.0001) and urothelial cancers (33.6%, p<0.0001). On the reverse, the prevalence of CKD was lower as compared to the average in 10 of the cancer groups but statistical significance was reached only in colonic cancer (5.3%, p = 0.0001) and brain tumor patients (2.5%, p = 0.006) “Table 3”. In fact, when multiple logistic regression was performed, the risk to develop CKD turned out to be significantly higher for eight cancer types (even when adjusted to age and gender) (“Table 4”). The increased prevalence of CKD in renal and urothelial cancers have been mentioned previously and it could be related to many specific therapeutic interventions (nephrectomy, invasion of the kidney, urinary tract obstructions, radiotherapy, etc.).

In our opinion, the real burden of CKD for any onco-nephrology team, in a specific area, is related to the absolute number of CKD cases (in some specific cancers) as well as the number of more advanced CKD patients (G4-G5).

In our area (our data base) the highest absolute number of CKD patients was registered among breast cancer, lung cancer and prostate cancer patients (“Table 3” and “Fig 1”). In fact, more than two third (67.8%) of all CKD cases are associated with only 7 cancer types (breast, lung, prostate, uterus, urinary tract, kidney and rectum).

The patients with CKD from our data base have been classified according to eGFR (stages G3 and higher) [5]. The majority (77.8%) were in CKD stage G3 (a-54.1% and b-23.7%), but the prevalence of more advanced stages was also higher than expected (G4–17% and G5 pre-dialysis—5.15%) “Table 2”. Data concerning prevalence of CKD stages in cancer and CKD patients are scarce and the authors used different classification types (or grouping of stages) [8, 9, 13, 14]. Nevertheless, our data suggest an increased prevalence of patients with more severe CKD (and at least one third of the stage 5 CKD patients having indication to initiate renal replacement therapy according to the cutoff values of GFR used in the IDEAL study). Explanation could be multiple: longer survival with cancer, more aggressive and repeated therapeutic intervention for relapses, more frequent AKI episodes, high prevalence of CKD at cancer diagnosis and inclusion in the data base and so on. Should be mentioned that cancer patients with end stage renal disease on hemodialysis treatment are not registered in our data base.

Our study has some advantages and limitations. The main advantage is the large number of cancer patients analyzed, a database representative for the Western Romanian population, which is very similar in characteristics with the Western Europe population. The cancer diagnosis is well documented and the lab results permitted diagnosis of CKD for the two years follow-up. The study has some important limitation also. Patients are not introduced in the data base at the cancer diagnosis time and site, therefore we could not analyze the impact of treatment on CKD development, initial kidney function were not available for assessment so we could not discuss the CKD prevalence before cancer diagnosis. The follow-up time is short therefore survival analysis could not be performed.

## Conclusions

The analysis of a large cancer data base from western Romania highlighted a significantly higher prevalence of CKD in these patients as compared to the general population. The prevalence was dependent of the age of the patients and the type of cancer being significantly higher in renal and urothelial cancer patients. The risk to develop CKD was significantly higher in some cancer types (renal, urinary tract, pharyngeal, pancreatic, prostatic, uterus and lung cancers) even when adjusted for age and gender, The prevalence of advanced CKD was surprisingly high (stages G4-G5-PD 22.15%), one third of the G5-Pre Dialysis patients having indication for initiation of renal replacement therapy. In our opinion, taking into account the high number of CKD cases in cancer patients the optimized management of these cases should include a nephrologist also.

## Supporting information

S1 Table Patients database. (XLSX)
